# Sex and APOE genotype modulate neuropsychological profile and depression in temporal lobe epilepsy

**DOI:** 10.3389/fnins.2024.1514902

**Published:** 2025-01-16

**Authors:** Francesco Bruno, Patrizia Spadafora, Ida Veltri, Mario L. Cuconati, Francesca Condino, Annamaria Cerantonio, Selene De Benedittis, Beatrice M. Greco, Gemma Di Palma, Olivier Gallo, Luigi Citrigno, Antonio Qualtieri, Maurizio Cundari, Francesca Cavalcanti

**Affiliations:** ^1^Faculty of Social and Communication Sciences, Universitas Mercatorum, Rome, Italy; ^2^Institute for Biomedical Research and Innovation (IRIB), Italian National Research Council (CNR), Cosenza, Italy; ^3^Territorial Social-Health Company of Lodi, Lodi, Italy; ^4^Department of Medical and Surgical Sciences, Science and Techniques of Cognitive Psychology Degree Course, Magna Graecia University of Catanzaro, Catanzaro, Italy; ^5^Department of Economics, Statistics and Finance “Giovanni Anania”, University of Calabria, Rende, Cosenza, Italy; ^6^Department of Biology, Ecology and Earth Sciences, University of Calabria, Rende, Cosenza, Italy; ^7^Department of Experimental Medical Science, Faculty of Medicine, Lund University, Lund, Sweden; ^8^Unit of Neuropsychiatry, Hospital of Helsingborg, Helsingborg, Sweden; ^9^Unit of Neurology, Hospital of Helsingborg, Helsingborg, Sweden

**Keywords:** temporal lobe epilepsy, APOE, sex, depression, neuropsychology, memory, executive functions, attention

## Abstract

**Introduction:**

Temporal lobe epilepsy is the most common form of focal epilepsy, often associated with cognitive impairments, particularly in memory functions, and depression. Sex and APOE ε4 genotype play a crucial role in modulating cognitive outcomes and depression in various neurological conditions like Alzheimer's disease. However, the combined effects of APOE genotype and sex on cognitive performance and depression in temporal lobe epilepsy have not been previously investigated.

**Objective:**

This study aims to (i) identify impaired cognitive performance and clinically relevant depression; (ii) explore the interaction between sex and APOE ε4 genotype on cognitive performance and depression in individuals with temporal lobe epilepsy.

**Methods:**

We used a comprehensive battery of neuropsychological tests to assess domains such as learning and memory, attention, executive functions, language, and visuo-spatial constructional skills and the Hamilton Depression Rating Scale. We also performed APOE genotyping to assess its role in the study. The final sample was composed by fifty-four patients (53.7% female). Cognitive performance and depression were analyzed using normative cut-off scores. To examine the main effects and interactions of sex and APOE ε4 carrier status on neuropsychological test scores and the Hamilton Depression Rating Scale, we also conducted a two-way Analysis of Variance (ANOVA).

**Results:**

Female APOE ε4 carriers compared to normative cut-offs, exhibited poor performance on multiple test scores, including the MMSE, The Rey Auditory Verbal Learning Test (immediate and delayed recall), The Corsi Block-Tapping Task, The Verbal Fluency Test, The Raven's Standard Progressive Matrices and The Pentagon-copying Test. Males showed impairment only in visuo-spatial short-term memory. ANOVA analysis revealed significant main effects of APOE ε4 status and sex on the MMSE, The Rey Auditory Verbal Learning Test, The Verbal Fluency, The Raven's Standard Progressive Matrices and The Pentagon-copying Test scores. Specifically, female APOE ε4 carriers performed consistently worse than other groups on many tasks. For depression, only an effect of sex emerged. Females scored higher besides APOE genotype.

**Conclusions:**

These findings underscore the importance of considering both sex and APOE genotype when assessing cognitive performance in patients with temporal lobe epilepsy. The significant cognitive deficits we observed among females carrying the APOE ε4 allele highlight previously unexplored genetic and sex-related influences on cognition. This has potential implications for personalized therapeutic strategies, emphasizing the need for targeted assessment and intervention.

## 1 Introduction

Temporal lobe epilepsy, the most common form of focal epilepsy, is characterized by recurrent, unprovoked seizures originating from the temporal lobes, particularly the hippocampus and surrounding structures (Engel, 1996; Téllez-Zenteno and Hernández-Ronquillo, 2012; Ladino et al., 2014). This condition is complex to diagnose and manage as it can present with a variety of clinical manifestations, including aura, automatisms, and impaired awareness during seizures. Furthermore, literature shows that it can also result in a range of cognitive alterations, particularly affecting memory, due to its impact on critical regions implicated in this process (Tramoni-Negre et al., 2017). Moreover, temporal lobe epilepsy is also associated with a high occurrence of depression (Garcia, 2012).

Apolipoprotein E (APOE) is a protein involved in lipid metabolism and neuronal repair, with its three major genotypes—ε2, ε3, and ε4—playing distinct roles in neurological health. It has long been known that there is a strong association between the ε4 allele and an increased risk of Alzheimer's disease as well as accelerated cognitive decline (Corder et al., 1993; Saunders et al., 1993). Recently, some researchers have extended the study of the APOE impact to other conditions, such as cardiovascular diseases (Abondio et al., 2023), traumatic brain injury (Lawrence et al., 2015), multiple sclerosis (Naseri et al., 2022) and temporal lobe epilepsy. In relation to the last one, it has been shown that APOE ε4 allele increases the risk (Liang et al., 2019; Kauffman et al., 2010) and is associated with an earlier onset of temporal lobe epilepsy (Kauffman et al., 2010). Moreover, Gambardella et al. (2005), Busch et al. (2007), and Han et al. (2024), underscored the role of the APOE ε4 genotype in exacerbating cognitive impairment in individuals with temporal lobe epilepsy.

Sex differences in cognitive impairment (Laws et al., 2016), as well as in the association between APOE ε4 genotype and cognitive impairment, have also been observed in other conditions (Beydoun et al., 2012; Makkar et al., 2020; Mortensen and Hogh, 2001). Specifically, in the context of Alzheimer's disease, studies have suggested that females have poorer cognitive performance compared to males (Laws et al., 2016). In addition, it has been shown that females carrying the APOE ε4 allele may experience more pronounced cognitive decline compared to males with the same genotype (Ungar et al., 2013; Gabelli and Codemo, 2015; Pike, 2016). Delano-Wood et al. (2008) reported that the presence of APOE ε4 allele predicts depression in females with Alzheimer's disease. This differential impact underscores the importance of considering both sex and genetic factors when assessing cognitive performance and depression in other neurological conditions.

In the present study, cognitive performance is compared between males and females with temporal lobe epilepsy, stratified by APOE ε4 carrier status, across several neuropsychological tests that assess general cognitive functioning, learning and memory, attention, executive functions, language, visuoconstructional abilities and visual-motor coordination. In addition, we compared the presence of depression between males and females taking into account the APOE genotype. We used normative cut-off scores to assess the degree of cognitive impairment and determine whether performance is within the normal range or indicative of impairment. Similarly, we evaluated depression levels to determine whether they fall above or below the cut-off. We also focused on comparing higher or lower raw scores across individuals, independent of their classification (cognitively impaired/depressed or not). This approach allows us to assess more subtle variations in cognitive performance and depression that may not necessarily reflect overt impairment but are still influenced by sex, APOE genotype, and their interaction.

## 2 Material and methods

### 2.1 Patients

The study included Italian patients of Caucasian (Mediterranean European) origin with non-lesional temporal lobe epilepsy followed at the National Research Council (CNR)—Institute for Biomedical Research and Innovation—(IRIB), (Mangone, Cosenza, Italy). All patients underwent a comprehensive clinical and neuropsychological evaluation to ensure the primary diagnosis of temporal lobe epilepsy. Data were retrospectively extracted from the respective medical records on the basis of completeness of clinical data. Inclusion criteria were: (i) age of more than 18 years; (ii) diagnosis of temporal lobe epilepsy according to the International Classification of Epilepsies, Epileptic Syndromes and Related Seizure Disorders (ILAE) classification of seizures (Commission on Classification and Terminology of the International League Against Epilepsy, 1989; Engel, 2001); (iii) presence of neuropsychological evaluation and Hamilton Depression Rating Scale score; (iv) MRI evidence of non-lesional epilepsy; (v) absence of a history of head injury, neurological illness other than epilepsy, intellectual disability or severe medical diseases; (vi) availability of a DNA sample. Individuals with a known history of progressive cognitive decline, concomitant neurodegenerative (e.g., Alzheimer's disease) or psychiatric disorders (e.g., Major Depressive Disorder) and drug-resistant epilepsy were excluded from the study. The work was done according to the Helsinki Declaration of 1975. Ethical review and approval were not required because the study involves the secondary use of non-identifiable information previously collected and anonymous biological materials, in accordance with the local legislation. Written consent for genetic screening was obtained from all participants.

### 2.2 Neuropsychological and mood evaluation

The cognitive functions of all patients were evaluated by a battery of standardized neuropsychological tests. The following tests were administered: (i) The **Mini-Mental State Examination** (MMSE) to assess general cognitive functioning (Magni et al., 1996); (ii) The **Rey Auditory Verbal Learning Test**—**immediate recall and delayed recall**, to evaluate verbal learning and memory (Carlesimo et al., 1996); (iii) The **Corsi Block-Tapping Task** to measure visuo-spatial short-term memory (De Renzi and Nichelli, 1975); (iv) The **Token Test** to evaluate receptive language abilities, particularly the comprehension of verbal instructions (Spinnler and Tognoni, 1987); (v) The **Phrase Construction Test** to assess several aspects of language production, including syntax and semantic coherence (Carlesimo et al., 1996); (vi) The **Verbal Fluency Test**, indicative of lexical access, the retrieval of terms from memory, and executive control (Carlesimo et al., 1996); (vii) The **Wisconsin Card Sorting Test (WCST)** to assess cognitive flexibility and perseverations. In particular, the “global score measure” represents an overall index of the WCST performance whereas the “perseverations measure” is useful to quantifies the perseverative behavior (Laiacona et al., 2000); (viii) The **Stroop Color and Word Test** to measure cognitive inhibition and selective attention with an interference procedure (Brugnolo et al., 2015); (ix) The **Raven's Standard Progressive Matrices** to evaluate non-verbal reasoning, abstract reasoning, and fluid intelligence (Caffarra et al., 2003); (x) The **Pentagon-copying Test** to assess visuoconstructive abilities and the capacity to coordinate visual and motor information (Caffarra et al., 2013). All tests were administered by following standardized procedures (Caltagirone et al., 1979), by examiners that were blind to the patients' electroclinical and genetic characteristics. The time required for the test administration was ~90 min. Normative-based cut-off scores were already available for all the tests (Magni et al., 1996; Carlesimo et al., 1996; De Renzi and Nichelli, 1975; Spinnler and Tognoni, 1987; Laiacona et al., 2000; Caffarra et al., 2003, 2013) except for the Pentagon-copying Test. In the last case we used as the reference value the mean obtained by patients with Alzheimer's disease (Caffarra et al., 2013). Moreover, depression was assessed using the Italian version of Hamilton Depression Rating Scale with a cut-off value of 6 (Fava et al., 1982; Mula et al., 2014).

### 2.3 APOE genotyping

Genomic DNA was extracted from peripheral venous blood using the “Salting Out” method (Miller et al., 1988). We amplified a 318 bp DNA fragment of the exon 4 of the APOE gene by Polymerase Chain Reaction (PCR) using the following primers: 5′ACTGACCCCGGTGGCGGAGGAGACGCGGGC-3 (F) and 5′TGTTCCACCAGGGGCCCCAGGCGCTCGCGG-3′ (R). APOE genotyping was performed using direct sequencing of rs429358 and rs7412 SNPs using the Big Dye Terminator Ready Reaction Mix v.3.1 and the ABI PRISM 3130 XL Genetic Analyzer (Applied Biosystems by Life Technologies).

### 2.4 Statistical analyses

Data were analyzed using Jamovi software (version 2.3.18). Descriptive statistics were conducted on demographic and clinical characteristics. Means and standard deviations (m ± SD) for continuous variables and frequencies and percentages (*n*/%) of categorical variables were generated. Differences between females and males for these variables were analyzed using *t*-tests or chi-square tests, depending on the type of variable. A two-way Analysis of Variance (ANOVA) was conducted to test the effects and the interactions of sex (i.e., male/female) and APOE genotype (i.e., APOE ε4 non-carriers/APOE ε4 carriers) on the scores of each neuropsychological test and Hamilton Depression Rating Scale. The effect size was estimated using omega squared (ω^2^). A *p*-value < 0.05 was considered statistically significant.

## 3 Results

Demographic and clinical characteristics of patients are reported in Table 1. The final sample consisted of 54 patients (53.7% females), with most participants being APOE ε4 non-carriers (88.9%). No significant differences were found between males and females regarding age (*p* = 0.110), age of onset (*p* = 0.104), duration of epilepsy (*p* = 0.759), years of education (*p* = 0.719), and use of antiepileptic drugs (*p* = 0.266). Regarding the latter, 12 females (99%) and 13 males (86.66%) used carbamazepine, one male (6.7%) and one female (1%) used gabapentin, and one male (6.7%) used vigabatrin.

**Table 1:** Demographic and clinical characteristics of the participants.

	**Female (*n* = 29)**	**Male (*n* =25)**	***P*-value**
Age	45.6 ± 16.4	53.7 ± 20.2	0.110
Age of onset	29.3 ± 17.4	38.7 ± 24.1	0.104
Duration of epilepsy (years)	16.3 ± 15.3	15.0 ± 15.3	0.759
Education (years)	7.90 ± 4.91	7.48 ± 3.24	0.719
Antiepiletic drugs use	13 (24.10)	15 (27.80)	0.266

Data are presented as mean ± standard deviation (m ± SD) or n (%).

Summary measures with relative cut-off scores are reported in Table 2. From the comparison between the scores obtained by the different groups (i.e., males and females APOE ε4 non-carriers and ε4 carriers) and the reference normative cut-off values, emerged that females APOE ε4 carriers obtained a poor performance on the **MMSE** (23.0 ± 1.7; cut-off ≤ 24), the **Rey Auditory Verbal Learning Test—immediate recall** (27.4 ± 4.9; cut-off ≤ 28.53), the **Rey Auditory Verbal Learning Test—delayed recall** (4.4 ± 2.6; cut-off ≤ 4.69), the **Corsi Block-tapping Task** (3.4 ± 1.2; cut-off ≤ 3.75), the **Verbal Fluency Test** (13.1 ± 6.5; cut-off ≤ 17.35), the **Stroop Color and Word Test** (346.0 ± 257.3; cut-off ≥ 257.83), the **Raven's Standard Progressive Matrices** (12.3 ± 10.0; cut-off ≤ 18.96) and the **Pentagon Coping Test** (6.00 ± 3.46; cut-off ≤ 9.35), whereas males obtained a poor performance on the **Corsi span-block** (3.1 ± 1.4; cut-off ≤ 3.75). Performance on all other neuropsychological tests were within normal cut-off values regardless of sex and APOE genotype. Moreover, for the **Hamilton Depression Rating Scale** emerged that females obtained an average score above the cut-off value besides APOE genotype (female APOE ε4 non-carriers = 7.56 ± 4.08; female APOE ε4 carriers = 7.66 ± 9.81; cut-off ≥ 6) (Lee et al., 2024).

**Table 2:** Mean scores obtained on each neuropsychological test by males and females based on APOE genotype.

	**Male**		**Female**		**Cut-off**
	ε**4–**	ε**4**+	ε**4–**	ε**4**+	
MMSE	27.45 ± 2.06	28.00 ± 1.00	28.16 ± 1.67	23.00 ± 1.73^*^	≤24
Rey Auditory Verbal Learning Test—immediate recall	35.75 ± 7.06	27.73 ± 11.89	40.01 ± 7.52	27.46 ± 4.92^*^	≤28.53
Rey Auditory Verbal Learning Test—delayed recall	7.35 ± 2.51	6.20 ± 2.33	8.67 ± 2.58	4.43 ± 2.63^*^	≤4.69
Corsi Block-Tapping Task	4.27 ± 1.48	3.16 ± 1.44^*^	4.38 ± 0.60	3.41 ± 1.23^*^	≤3.75
Token Test	31.10 ± 3.75	33.08 ± 1.37	32.82 ± 2.10	29.41 ± 6.86	≤26.5
Phrase Construction Test	19.11 ± 5.95	22.20 ± 2.16	19.71 ± 5.18	17.56 ± 7.14	≤8.72
Verbal Fluency Test	24.55 ± 10.74	18.53 ± 4.47	24.70 ± 7.51	13.13 ± 6.55^*^	≤17.35
WCST—Global Score	63.01 ± 33.69	51.63 ± 40.12	43.06 ± 25.67	73.35 ± 64.13	≥90.6
WCST—Perseverations	22.99 ± 20.01	22.86 ± 15.70	11.22 ± 10.73	37.93 ± 49.30	≥42.7
Stroop Color and Word Test	158.33 ± 21.57	232.05 ± 101.87	180.49 ± 53.27	346.00 ± 257.38^*^	≥257.83
Raven's Standard Progressive Matrices	26.22 ± 7.99	29.00 ± 8.88	28.53 ± 6.26	12.33 ± 10.01^*^	≤20.72
Pentagon-Copying Test	11.10 ± 1.77	11.00 ± 1.73	11.08 ± 2.16	6.00 ± 3.46^*^	≤9.35
Hamilton Depression Rating Scale	4.09 ± 2.34	2.66 ± 4.61	7.56 ± 4.08^*^	7.66 ± 9.81^*^	≥6

Data are presented as mean ± standard deviation (m ± SD).

ε4–, APOE ε4 non-carriers; ε4+, APOE ε4 carriers.

* Under cut-off values.

The results of the two-way ANOVA analysis are reported in Table 3. About the **MMSE**, the analysis showed a significant main of sex (*F* = 7.31, df = 1, *p* = 0.010, ω^2^ = 0.082)—i.e., females obtained lower MMSE score compared to males: estimated marginal mean (EMM) males = 27.7 ± 0.565; EMM females = 25.6 ± 0.557—APOE genotype (*F* = 8.45, df = 1, *p* = 0.006, ω^2^ = 0.097)—i.e., APOE ε4 carriers obtained MMSE lower scores compared to APOE ε4 non-carriers (EMM ε4 non-carriers = 27.8 ± 0.274; EMM APOE ε4 carriers = 25.5 ± 0.745)—and of sex/APOE interaction (*F* = 12.96, df = 1, *p* = 0.001, ω^2^ = 0.156)—i.e., females APOE ε4 carriers obtained lower MMSE scores compared to the other groups (EMM males APOE ε4 non-carriers = 27.4 ± 0.408; EMM males APOE ε4 carriers = 28.0 ± 1.053; EMM females APOE ε4 non-carriers = 28.2 ± 0.365; EMM females APOE ε4 carriers = 23.0 ± 1.053). Regarding the **Rey Auditory Verbal Learning Test—immediate recall** the analysis showed a significant main effect of APOE genotype (*F* = 10.079, df = 1, *p* = 0.003, ω^2^ = 0.147), indicating that APOE ε4 carriers obtained lower scores compared to APOE ε4 non-carriers (EMM ε4 non-carriers = 37.9 ± 1.08; APOE ε4 carriers = 27.6 ± 3.05). About the **Rey Auditory Verbal Learning Test—delayed recall** the analysis showed a significant main effect of APOE genotype (*F* = 5.9993, df = 1, *p* = 0.018, ω^2^ = 0.085), indicating that APOE ε4 carriers obtained lower scores compared to APOE ε4 non-carriers (EMM APOE ε4 non-carriers = 8.02 ± 0.369; EMM APOE ε4 carriers = 5.32 ± 1.039). Considering the **Corsi Block-tapping Task**, the analysis showed a significant main effect of APOE genotype (*F* = 4.5682, df = 1, *p* = 0.037, ω^2^ = 0.064), indicating that APOE ε4 carriers obtained lower scores compared to APOE ε4 non-carriers (EMM APOE ε4 non-carriers = 4.33 ± 0.162; EMM ε4 carriers = 3.29 ± 0.457). About the **Verbal Fluency Test**, emerged a significant main effect of APOE genotype (*F* = 5.204, df = 1, *p* = 0.027, ω^2^ = 0.074), indicating that APOE ε4 carriers obtained lower scores compared to APOE ε4 non-carriers (EMM ε4 non-carriers = 24.6 ± 1.29; EMM APOE ε4 carriers = 15.8 ± 3.63). Regarding the **Raven's Standard Progressive Matrices** the analysis showed a significant main of sex (*F* = 5.12, df = 1, *p* = 0.028, ω^2^ = 0.059)—i.e., females obtained lower scores compared to males (EMM males = 27.6 ± 2.25; EMM females = 20.4 ± 2.23)—APOE genotype (*F* = 4.48, df = 1, *p* = 0.039, ω^2^ = 0.050) —i.e., APOE ε4 carriers obtained lower scores compared to APOE ε4 non-carriers (EMM APOE ε4 non-carriers = 27.4 ± 1.06; EMM ε4 carriers = 20.7 ± 2.99)—and of sex/APOE interaction (*F* = 8.94, df = 1, *p* = 0.004, ω^2^ = 0.114)—i.e., females APOE ε4 carriers obtained lower scores compared to the other groups (EMM males APOE ε4 non-carriers = 26.2 ± 1.56; EMM males APOE ε4 carriers = 29.0 ± 4.23; EMM females APOE ε4 non-carriers = 28.5 ± 1.44; EMM female APOE ε4 carriers = 12.3 ± 4.23). Considering the **Stroop Color and Word Test** the analysis showed a significant main of sex/APOE interaction (*F* = 8.59, df = 1, *p* = 0.005, ω^2^ = 0.121)—i.e., females APOE ε4 carriers obtained lower scores compared to the other groups (EMM males APOE ε4 non-carriers = 232 ± 18.2; EMM males APOE ε4 carriers = 158 ± 49.2; EMM females APOE ε4 non-carriers= 180 ± 16.7; EMM female APOE ε4 carriers = 346 ± 60.3). Regarding the **Pentagon-copy Test** the analysis showed a significant main of sex (*F* = 8.67, df = 1, *p* = 0.005, ω^2^= 0.107)—i.e., females obtained lower scores compared to males (EMM males = 11.22 ± 0.646; EMM females = 8.54 ± 0.639)—APOE genotype (*F* = 7.12, df = 1, *p* = 0.010, ω^2^= 0.086)—i.e., APOE ε4 carriers obtained lower scores compared to APOE ε4 non-carriers (EMM APOE ε4 non-carriers = 11.09 ± 0.316; EMM ε4 carriers = 8.67 ± 0.852)—and of sex/APOE interaction (*F* = 8.56, df = 1, *p* = 0.005, ω^2^ = 0.106)—i.e., females APOE ε4 carriers obtained lower scores compared to the other groups (EMM males APOE ε4 non-carriers = 11.10 ± 0.467; EMM males APOE ε4 carriers = 11.33 ± 1.205; EMM females APOE ε4 non-carriers = 11.08 ± 0.426; EMM female APOE ε4 carriers = 6.0 ± 1.205). No statistically significant results emerged for the **Token Test, Phrase Construction Test, WCST—global score and WCST – perseverations**. Finally, for the **Hamilton Depression Rating Scale** the analysis showed a significant main of sex (*F* = 4.838, df = 1, *p* = 0.033, ω^2^= 0.069)—i.e., females obtained higher scores compared to males (EMM males = 3.38 ± 1.10; EMM females = 6.80 ± 1.10).

**Table 3:** Results of a two-way ANOVA examining the effects of sex, APOE genotype, and their interaction on neuropsychological performance and depression.

	**Sum of squares**	**df**	***F***	***P*-value**	**ω^2^**
**MMSE**					
Sex	24.3	1	7.31	0.010^**^	0.082
APOE	28.1	1	8.45	0.006^**^	0.097
Sex ^*^ APOE	43.1	1	12.96	0.001^**^	0.156
**Rey Auditory Verbal Learning Test—immediate recall**					
Sex	21.2	1	0.379	0.541	−0.010
APOE	563.5	1	10.079	0.003^**^	0.147
Sex ^*^ APOE	27.3	1	0.488	0.488	−0.008
**Rey Auditory Verbal Learning Test—delayed recall**					
Sex	0.268	1	0.0414	0.840	−0.016
APOE	38.888	1	5.9993	0.018^*^	0.085
Sex ^*^ APOE	12.676	1	1.9555	0.168	0.016
**Corsi Block-tapping Task**					
Sex	0.1745	1	0.1391	0.711	−0.015
APOE	5.7309	1	4.5682	0.037^*^	0.064
Sex ^*^ APOE	0.0254	1	0.0203	0.887	−0.018
**Token Test**					
Sex	5.02	1	0.498	0.484	−0.009
APOE	2.72	1	0.270	0.606	−0.013
Sex ^*^ APOE	38.72	1	3.839	0.056	0.051
**Phrase Construction Test**					
Sex	21.74	1	0.7120	0.403	−0.005
APOE	1.17	1	0.0383	0.846	−0.018
Sex ^*^ APOE	36.40	1	1.1918	0.280	0.004
**Verbal Fluency Test**					
Sex	36.7	1	0.463	0.499	−0.009
APOE	412.1	1	5.204	0.027^*^	0.074
Sex ^*^ APOE	41.1	1	0.519	0.475	−0.008
**WCST—Global Score**					
Sex	3.32	1	0.00326	0.955	−0.024
APOE	380.76	1	0.37462	0.544	−0.015
Sex ^*^ APOE	1,849.92	1	1.82009	0.185	0.019
**WCST—Perseverations**					
Sex	14.2	1	0.0393	0.844	−0.020
APOE	922.2	1	2.5561	0.117	0.032
Sex ^*^ APOE	939.4	1	2.6038	0.114	0.033
**Stroop Color and Word Test**					
Sex	20,197	1	2.78	0.102	0.028
APOE	9,185	1	1.26	0.266	0.004
Sex ^*^ APOE	62,387	1	8.59	0.005^**^	0.121
**Raven's Standard Progressive Matrices**					
Sex	275	1	5.12	0.028^*^	0.059
APOE	240	1	4.48	0.039^*^	0.050
Sex ^*^ APOE	480	1	8.94	0.004^**^	0.114
**Pentagon-Copying Test**					
Sex	37.7	1	8.67	0.005^**^	0.107
APOE	31.0	1	7.12	0.010^**^	0.086
Sex ^*^ APOE	37.3	1	8.56	0.005^**^	0.106
**Hamilton Depression Rating Scale**					
Sex	62.25	1	4.838	0.033^*^	0.069
APOE	1.39	1	0.108	0.743	−0.016
Sex ^*^ APOE	4.42	1	0.344	0.560	−0.012

Sex, male/female; APOE, APOE ε4 non-carries/APOE ε4 carries; df, degree of freedom.

* Significant at 0.05 level.

** Significant at 0.01 level.

## 4 Discussion

The findings from this study reveal significant interactions between sex, APOE ε4 genotype, cognitive performance and depression in individuals with temporal lobe epilepsy. Our study adopted a dual methodological approach: comparing cognitive test and depression scores to normative cut-off values and performing ANOVA to explore the main effects and interactions between sex and APOE genotype on cognitive outcomes and depression. This dual approach allowed for a comprehensive understanding of cognitive outcomes and depression in temporal lobe epilepsy, revealing distinct patterns of cognitive deficits and depression associated with sex and APOE genotype.

From the comparison of cognitive test scores to normative cut-off values emerged that females APOE ε4 carriers obtained a poor performance on tests that assessed general cognitive functioning (i.e., the MMSE), verbal learning and memory (i.e., the Rey Auditory Verbal Learning Test—immediate and delayed recall), short-term visuo-spatial memory (i.e., the Corsi Block-Tapping Task), lexical access and executive control (i.e., the Verbal Fluency Test), cognitive inhibition and selective attention (i.e., the Stroop Color and Word Test), non-verbal reasoning, abstract reasoning, and fluid intelligence (i.e., the Raven's Standard Progressive Matrices), visuoconstructive abilities and the capacity to coordinate visual and motor information (i.e., the Pentagon-Copying Test). This finding aligns with previous research that highlights the exacerbated cognitive decline associated with the APOE ε4 allele in females (Mortensen and Hogh, 2001), particularly within the context of neurodegenerative conditions such as Alzheimer's disease (Ungar et al., 2013). The notion that females are more vulnerable to cognitive impairment in the presence of the APOE ε4 genotype has been supported by studies indicating that sex differences can significantly modulate the cognitive effects of genetic risk factors (Duarte-Guterman et al., 2021). Conversely, males exhibited only cognitive deficits in short-term visuo-spatial memory (i.e., the Corsi Block-Tapping Task), and thus the overall impact of the APOE ε4 allele on cognitive performance appeared less pronounced in this group. This suggests a potential protective effect in males that warrants further investigation, as sex differences in cognitive aging and neurological conditions are increasingly recognized. For instance, it has been suggested that these female-specific effects may be mediated by sex hormones, which could amplify the negative impact of the APOE ε4 allele (Walters et al., 2023). Interestingly, Saleh et al. (2023) explored the effects of hormone replacement therapy (HRT) on cognition and brain structure in females who are carriers of the APOE ε4 allele, which increases the risk of Alzheimer's disease. Using data from the European Prevention of Alzheimer's Dementia (EPAD) cohort, the study found that APOE ε4 carriers who used HRT had improved delayed memory performance and larger brain volumes, specifically in the entorhinal cortex and amygdala, compared to non-users and non-carriers. Furthermore, initiating HRT earlier was associated with larger hippocampal volumes in APOE ε4 carriers, suggesting a potential neuroprotective effect. These findings suggest that HRT could mitigate some of the cognitive decline associated with APOE ε4 in females, but further randomized controlled trials are needed to confirm these results and establish causality.

In addition to cognitive performance, our study found that females consistently scored higher on the Hamilton Depression Rating Scale, indicating a greater prevalence of depression irrespective of their APOE genotype. This finding is consistent with existing literature that highlights a higher incidence of depression in females with epilepsy (Gaus et al., 2015), potentially attributable to a complex interplay of biological, psychological, and social factors (Remes et al., 2021).

From the ANOVA analysis—performed to explore the main effects and interactions between sex and APOE genotype on cognitive outcomes and depression—emerged significant results that complement the findings from the cut-off comparisons. Specifically, the ANOVA revealed that the interaction between sex and APOE ε4 status significantly influenced cognitive performance, suggesting that the impact of the APOE ε4 allele is not uniform across sexes. Females who are carriers of the APOE ε4 allele exhibited the most pronounced cognitive deficits compared to their male counterparts and non-carriers. This supports the findings from the cut-off comparisons, where female carriers consistently demonstrated lower scores across various neuropsychological tests, confirming their heightened vulnerability to cognitive deficits linked to the APOE ε4 genotype.

It is noteworthy that the performance on the WCST (i.e., cognitive flexibility and perseverations), the Token Test (i.e., receptive language abilities and comprehension of verbal instructions), and The Phrase Construction Test (i.e., the ability to construct meaningful sentences from given words) were not influenced by sex and/or APOE genotype. The lack of observed effects on these tests suggests that the cognitive functions they assess may be less susceptible to the influence of APOE ε4 genotype and sex compared to other ones. Furthermore, depression scores followed a similar trend; ANOVA analysis indicated a significant interaction effect where female APOE ε4 carriers exhibited the highest depression levels, corroborating the previous findings that showed females scoring higher on the Hamilton Depression Rating Scale. This reinforces the notion that female patients with temporal lobe epilepsy and the APOE ε4 allele are at an increased risk for both cognitive decline and depression. Overall, the ANOVA findings enhance our understanding of the nuanced relationships between sex, genetic predisposition, and cognitive and emotional outcomes in this population. By demonstrating that both cognitive performance and depressive symptoms are significantly impacted by the interplay between sex and APOE genotype, we highlight the need for tailored interventions that address these disparities.

However, despite the interesting results, this study has several limitations that must be considered. First, the sample size, while adequate for preliminary analysis, is relatively small and may not adequately represent the broader population of patients with temporal lobe epilepsy. Second, the retrospective design of our study means that data were extracted from existing medical records, which may lead to the omission of relevant information or recording errors. For instance, regarding patients on antiepileptic drug treatment, we did not have information on the duration of treatment and thus, whether there were any differences between males and females in this regard. Future studies should address this aspect. Additionally, the lack of longitudinal assessments restricts our understanding of cognitive changes over time. Furthermore, the analysis primarily focused on a specific set of neuropsychological tests, which may not have captured all aspects of cognitive domains. Moreover, due to the small sample size, we were only able to analyze the effects of APOE ε4 carriers and non-carriers, not the other APOE genotypes. Future studies should consider investigating the effects of different APOE genotypes on neuropsychological profile and depression in temporal lobe epilepsy.

## 5 Conclusion

In summary, our study provides valuable insights into the complex interplay between sex, APOE genotype, and cognitive performance in temporal lobe epilepsy. The identification of broader cognitive deficits, particularly in females with APOE ε4, highlights the need for targeted assessment and intervention strategies. Future research should continue to explore these interactions and their implications for both clinical practice and our understanding of cognitive deficits in temporal lobe epilepsy and related neurological conditions.

## Data Availability

The data analyzed in this study is subject to the following licenses/restrictions: the raw data supporting the conclusions of this article will be made available by the authors, without undue reservation. Requests to access these datasets should be directed to francesca.cavalcanti@irib.cnr.it.

## References

[B1] Abondio P. Bruno F. Luiselli D. (2023). Apolipoprotein E (APOE) haplotypes in healthy subjects from worldwide macroareas: a population genetics perspective for cardiovascular disease, neurodegeneration, and dementia. Curr. Issues Mol. Biol. 45, 2817–2831. 10.3390/cimb45040184 37185708 PMC10137191

[B2] Beydoun M. A. Boueiz A. Abougergi M. S. Kitner-Triolo M. H. Beydoun H. A. Resnick S. M.. (2012). Sex differences in the association of the apolipoprotein E epsilon 4 allele with incidence of dementia, cognitive impairment, and decline. Neurobiol. Aging 33, 720–731. 10.1016/j.neurobiolaging.2010.05.017 20619505 PMC2974952

[B3] Brugnolo A. De Carli F. Accardo J. Amore M. Bosia L. E. Bruzzaniti C.. (2015). An updated Italian normative dataset for the Stroop color word test (SCWT). Neurol. Sci. 37, 365–372. 10.1007/s10072-015-2428-2 26621362

[B4] Busch R. M. Lineweaver T. T. Naugle R. I. Kim K. H. Gong Y. Tilelli C. Q.. (2007). ApoE-ε4 is associated with reduced memory in long-standing intractable temporal lobe epilepsy. Neurology 68, 409–414. 10.1212/01.wnl.0000253021.60887.db 17283313

[B5] Caffarra P. Gardini S. Dieci F. Copelli S. L. Maset L. Concari L.. (2013). The qualitative scoring MMSE pentagon test (QSPT): a new method for differentiating dementia with Lewy Body from Alzheimer's disease. Behav. Neurol. 27, 213–220. 10.1155/2013/728158 23396218 PMC5215738

[B6] Caffarra P. Vezzadini G. Zonato F. Copelli S. Venneri A. (2003). A normative study of a shorter version of Raven's progressive matrices 1938. Neurol. Sci. 24, 336–339. 10.1007/s10072-003-0185-0 14716529

[B7] Caltagirone C. Gainotti G. Masullo C. Miceli G. (1979). Validity of some neuropsychological tests in the assessment of mental deterioration. Acta Psychiatr. Scand. 60, 50–56. 10.1111/j.1600-0447.1979.tb00264.x 474177

[B8] Carlesimo G. A. Caltagirone C. Gainotti G. Fadda L. Gallassi R. Lorusso S.. (1996). The mental deterioration battery: normative data, diagnostic reliability and qualitative analyses of cognitive impairment. Eur. Neurol. 36, 378–384. 10.1159/000117297 8954307

[B9] Commission on Classification and Terminology of the International League Against Epilepsy (1989). Proposal for revised classification of epilepsies and epileptic syndromes. Epilepsia 30, 389–399. 10.1111/j.1528-1157.1989.tb05316.x 2502382

[B10] Corder E. Saunders A. Strittmatter W. Schmechel D. Gaskell P. Small G.. (1993). Gene dose of apolipoprotein E type 4 allele and the risk of Alzheimer's disease in late onset families. Science 261, 921–923. 10.1126/science.8346443 8346443

[B11] De Renzi E. Nichelli P. (1975). Verbal and non-verbal short-term memory impairment following hemispheric damage. Cortex 11, 341–354. 10.1016/S0010-9452(75)80026-8 1222578

[B12] Delano-Wood L. Houston W. S. Emond J. A. Marchant N. L. Salmon D. P. Jeste D. V.. (2008). APOE genotype predicts depression in women with Alzheimer's disease: a retrospective study. Int. J. Geriatr. Psychiatry 23, 632–636. 10.1002/gps.1953 18058831 PMC2583456

[B13] Duarte-Guterman P. Albert A. Y. Barha C. K. Galea L. A. M. on behalf of the Alzheimer's Disease Neuroimaging Initiative (2021). Sex influences the effects of APOE genotype and Alzheimer's diagnosis on neuropathology and memory. Psychoneuroendocrinology 129:105248. 10.1016/j.psyneuen.2021.105248 33962245

[B14] Engel J. (1996). Introduction to temporal lobe epilepsy. Epilepsy Res. 26, 141–150. 10.1016/S0920-1211(96)00043-5 8985696

[B15] Engel J. (2001). A proposed diagnostic scheme for people with epileptic seizures and with epilepsy: report of the ILAE Task Force on Classification and Terminology. Epilepsia 42, 796–803. 10.1046/j.1528-1157.2001.10401.x 11422340

[B16] Fava G. A. Kellner R. Munari F. Pavan L. (1982). The Hamilton Depression Rating Scale in normals and depressives. Acta Psychiatr. Scand. 66, 26–32. 10.1111/j.1600-0447.1982.tb00911.x 7124430

[B17] Gabelli C. Codemo A. (2015). Gender differences in cognitive decline and Alzheimer's disease. Ital. J. Gender Specific Med. 1, 21–28.

[B18] Gambardella A. Aguglia U. Chifari R. Labate A. Manna I. Serra P.. (2005). ApoE Epsilon4 allele and disease duration affect verbal learning in mild temporal lobe epilepsy. Epilepsia 46, 110–117. 10.1111/j.0013-9580.2005.15804.x 15660776

[B19] Garcia C. S. (2012). Depression in temporal lobe epilepsy: a review of prevalence, clinical features, and management considerations. Epilepsy Res. Treat. 2012:809843. 10.1155/2012/809843 22957244 PMC3420378

[B20] Gaus V. Kiep H. Holtkamp M. Burkert S. Kendel F. (2015). Gender differences in depression, but not in anxiety in people with epilepsy. Seizure 32, 37–42. 10.1016/j.seizure.2015.07.012 26552559

[B21] Han Y. Hao G. Wang Z. Wang C. Qi X. Liang G.. (2024). Association between serum apolipoprotein E and cognitive function in Chinese patients with temporal lobe epilepsy. Epilepsy Behav. 154, 109750–109750. 10.1016/j.yebeh.2024.109750 38552413

[B22] Kauffman M. A. Consalvo D. Moron D. G. Lereis V. P. Kochen S. (2010). ApoE ε4 genotype and the age at onset of temporal lobe epilepsy: a case–control study and meta-analysis. Epilepsy Res. 90, 234–239. 10.1016/j.eplepsyres.2010.05.007 20554432

[B23] Ladino L. D. Moien-Afshari F. Téllez-Zenteno J. F. (2014). “A comprehensive review of temporal lobe epilepsy,” in Neurological Disorders. Clinical Methods Edition, 1st Edn (iConcept Press Ltd), 1–35.

[B24] Laiacona M. Inzaghi M. G. De Tanti A. Capitani E. (2000). Wisconsin card sorting test: a new global score, with Italian norms, and its relationship with the Weigl sorting test. Neurol. Sci. 21, 279–291. 10.1007/s100720070065 11286040

[B25] Lawrence D. W. Comper P. Hutchison M. G. Sharma B. (2015). The role of apolipoprotein E episilon (ε)-4 allele on outcome following traumatic brain injury: a systematic review. Brain Inj. 29, 1018–1031. 10.3109/02699052.2015.1005131 25915580

[B26] Laws K. R. Irvine K. Gale T. M. (2016). Sex differences in cognitive impairment in Alzheimer's disease. World J. Psychiatry 6:54. 10.5498/wjp.v6.i1.54 27014598 PMC4804268

[B27] Lee M. Y. Zhu J. D. Tsai H. J. Tsai S. J. Yang A. C. (2024). Investigating sex-related differences in brain structure and function in bipolar I disorder using multimodal MRI. BMC Psychiatry 24:855. 10.1186/s12888-024-06228-7 39604920 PMC11603873

[B28] Liang Y. Zhou Z. Wang H. Cheng X. Zhong S. Zhao C. (2019). Association of apolipoprotein E genotypes with epilepsy risk: A systematic review and meta-analysis. Epilepsy Behav. 98, 27–35. 10.1016/j.yebeh.2019.06.015 31299529

[B29] Magni E. Binetti G. Bianchetti A. Rozzini R. Trabucchi M. (1996). Mini-Mental State Examination: a normative study in Italian elderly population. Eur. J. Neurol. 3, 198–202. 10.1111/j.1468-1331.1996.tb00423.x 21284770

[B30] Makkar S. R. Lipnicki D. M. Crawford J. D. Kochan N. A. Castro-Costa E. Lima-Costa M. F.. (2020). APOE ε4 and the influence of sex, age, vascular risk factors, and ethnicity on cognitive decline. J. Gerontol. Ser. A 75, 1863–1873. 10.1093/gerona/glaa116 32396611 PMC7518559

[B31] Miller S. A. Dykes D. D. Polesky H. F. (1988). A simple salting out procedure for extracting DNA from human nucleated cells. Nucl. Acids Res. 16, 1215–1215. 10.1093/nar/16.3.1215 3344216 PMC334765

[B32] Mortensen E. L. Hogh P. (2001). A gender difference in the association between APOE genotype and age-related cognitive decline. Neurology 57, 89–95. 10.1212/WNL.57.1.89 11445633

[B33] Mula M. Iudice A. La Neve A. Mazza M. Mazza S. Cantello R.. (2014). Validation of the Hamilton Rating Scale for Depression in adults with epilepsy. Epilep. Behav. 41, 122–125. 10.1016/j.yebeh.2014.08.029 25461202

[B34] Naseri A. Baghernezhad K. Seyedi-Sahebari S. Alhoseini S. A. Gholipour-khalili E. Zafarani F.. (2022). The association of apolipoprotein E (ApoE) genotype and cognitive outcomes in multiple sclerosis; a systematic review and meta-analysis. Mult. Scler. Relat. Disord. 65:104011. 10.1016/j.msard.2022.104011 35803087

[B35] Pike C. J. (2016). Sex and the development of Alzheimer's disease. J. Neurosci. Res. 95, 671–680. 10.1002/jnr.23827 27870425 PMC5120614

[B36] Remes O. Mendes J. F. Templeton P. (2021). Biological, psychological, and social determinants of depression: a review of recent literature. Brain Sci. 11, 1–33. 10.3390/brainsci11121633 34942936 PMC8699555

[B37] Saleh R. N. M. Hornberger M. Ritchie C. W. Minihane A. M. (2023). Hormone replacement therapy is associated with improved cognition and larger brain volumes in at-risk APOE4 women: results from the European Prevention of Alzheimer's Disease (EPAD) cohort. Alzheimers Res. Therapy 15:10. 10.1186/s13195-022-01121-5 36624497 PMC9830747

[B38] Saunders A. M. Strittmatter W. J. Schmechel D. St. George-Hyslop P. H. Pericak-Vance M. A. Joo S. H.. (1993). Association of apolipoprotein E allele 4 with late-onset familial and sporadic Alzheimer's disease. Neurology 43, 1467–1467. 10.1212/WNL.43.8.1467 8350998

[B39] Spinnler H. Tognoni G. (1987). Standardizzazione e taratura italiana dei test neuropsicologici. Italy J. Neurol. Sci. 6, 8–20.

[B40] Téllez-Zenteno J. F. Hernández-Ronquillo L. (2012). A review of the epidemiology of temporal lobe epilepsy. Epilepsy Res. Treat. 2012, 1–5. 10.1155/2012/630853 22957234 PMC3420432

[B41] Tramoni-Negre E. Lambert I. Bartolomei F. Felician O. (2017). Long-term memory deficits in temporal lobe epilepsy. Rev. Neurol. 173, 490–497. 10.1016/j.neurol.2017.06.011 28838789

[B42] Ungar L. Altmann A. Greicius M. D. (2013). Apolipoprotein E, gender, and Alzheimer's disease: an overlooked, but potent and promising interaction. Brain Imaging Behav. 8, 262–273. 10.1007/s11682-013-9272-x 24293121 PMC4282773

[B43] Walters S. Contreras A. G. Eissman J. M. Mukherjee S. Lee M. L. Choi S.. (2023). Associations of sex, race, and apolipoprotein E alleles with multiple domains of cognition among older adults. JAMA Neurol. 80, 929–929. 10.1001/jamaneurol.2023.2169 37459083 PMC10352930

